# Mobilized Peripheral Blood versus Cord Blood: Insight into the Distinct Role of Proinflammatory Cytokines on Survival, Clonogenic Ability, and Migration of CD34^+^ Cells

**DOI:** 10.1155/2018/5974613

**Published:** 2018-07-04

**Authors:** Dorian Forte, Daria Sollazzo, Martina Barone, Marisole Allegri, Angela di Martella Orsi, Marco Romano, Barbara Sinigaglia, Giuseppe Auteri, Nicola Vianelli, Michele Cavo, Francesca Palandri, Lucia Catani

**Affiliations:** ^1^Department of Experimental, Diagnostic and Specialty Medicine, University of Bologna, Bologna, Italy; ^2^Wellcome Trust-Medical Research Council Cambridge Stem Cell Institute and Department of Haematology, University of Cambridge and National Health Service Blood and Transplant, Cambridge Biomedical Campus, CB2 0PT Cambridge, UK; ^3^School of Immunology & Microbial Sciences, King's College London, Guy's Hospital, SE1 9RT London, UK

## Abstract

Inflammation may play a role in cancer. However, the contribution of cytokine-mediated crosstalk between normal hemopoietic stem/progenitor cells (HSPCs) and their (inflammatory) microenvironment is largely elusive. Here we compared survival, phenotype, and function of neonatal (umbilical cord blood (CB)) and adult (normal G-CSF-mobilized peripheral blood (mPB)) CD34^+^ cells after *in vitro* exposure to combined crucial inflammatory factors such as interleukin- (IL-) 1*β*, IL-6, tumor necrosis factor- (TNF-) *α*, or tissue inhibitor of metalloproteinases-1 (TIMP-1). To mimic bone marrow (BM) niche, coculture experiments with normal BM stromal cells (BMSCs) were also performed. We found that combined inflammatory cytokines increased only the *in vitro* survival of CB-derived CD34^+^ cells by reducing apoptosis. Conversely, selected combinations of inflammatory cytokines (IL-1*β* + TNF-*α*, IL-6 + TNF-*α*, and IL-1*β* + TNF-*α* + TIMP-1) mainly enhanced the *in vitro* CXCR4-driven migration of mPB-derived CD34^+^ cells. TNF-*α*, alone or in combination, upregulated CD44 and CD13 expression in both sources. Finally, BMSCs alone increased survival/migration of CB- and mPB-derived CD34^+^ cells at the same extent of the combined inflammatory cytokines; importantly, their copresence did not show additive/synergistic effect. Taken together, these data indicate that combined proinflammatory stimuli promote distinct *in vitro* functional activation of neonatal or adult normal HSPCs.

## 1. Introduction

Hemopoietic stem/progenitor cell (HSPC) activation and retention are modulated by the bone marrow (BM) niche where they are located. In response to inflammation and/or BM injury, long-term quiescent hemopoietic stem cells (HSCs) are efficiently recruited into the cell cycle progression returning back to quiescence after reestablishment of homeostasis [1, 2]. Inflammation is a fundamental response that protects tissues from damage and preserves internal homeostasis. However, chronic inflammation may hinder functionality of different tissues and has been suggested to cover a key role in cancer [3].

Proinflammatory cytokines are emerging as key regulators of steady-state and infection-driven hemopoiesis. Recent findings contributed to highlight how HSPC fate could be dictated by inflammatory factors in the BM microenvironment as HSPCs may actively respond to danger signals and proinflammatory cytokines [4, 5]. However, excessive chronic signalling can have negative effects on HSPC regulation and function [6]. Moreover, abnormalities in the inflammatory signalling pathways have been discovered in both preleukemic and leukemic diseases [7]. BM mesenchymal stromal cells (BMSCs) are one of the most important components of the BM microenvironment. They respond to various microenvironment stimuli by changing their secretory capacity and displaying immune-suppressive activity through direct or indirect production of prostaglandin E-2, indoleamine 2,3-dioxygenase, interleukin- (IL-) 10 [8–10], and soluble receptors for IL-1 and tumor necrosis factor-*α* (TNF-*α*) [11]. However, a crosstalk between HSPCs and the stromal cells may also create a proinflammatory environment that promotes malignant transformation and disease progression [12]. In such process, several factors and pathways have been implicated but it is not clear how inflammation could affect or transform HSPCs. Understanding the direct cellular target(s) of proinflammatory cytokines is a critical step to better clarifying how HSCs/HSPCs are regulated in the BM niche.

Granulocyte colony-stimulating factor- (G-CSF-) mobilized peripheral blood (mPB) and umbilical cord blood (CB) are two of the current sources of HSPCs for transplantation in hematological malignancies [13]; however, insights into the effects mediated by inflammation on neonatal and adult HSPCs are still elusive. In the last years, several phenotypic and functional differences between CB and mPB-derived HSPCs have been described [14–19]. However, so far, studies analyzing the adaptations of HSPCs from these two sources to inflammatory cytokines were focused on a limited number of cytokines which were individually tested [20–24].

To mirror the *in vivo* inflammatory microenvironment, here we investigated the role of combined crucial proinflammatory cytokines (IL-1*β*, TNF-*α*, IL-6, and tissue inhibitor of metalloproteases (TIMP-1)) on the *in vitro* functional behavior of CB- or mPB-derived CD34^+^ cells in the presence or absence of BMSCs.

## 2. Materials and Methods

### 2.1. Sample Collection

CB samples (*n* = 14) from normal full-term deliveries were provided by the Cord Blood Bank of the University Hospital of Bologna after written informed consent. mPB samples (*n* = 14) were obtained from hemopoietic stem cell transplantation donors. This study was approved by the medical Ethical Committee of the University Hospital of Bologna and was conducted in accordance with the Declaration of Helsinki.

### 2.2. Cell Isolation

Mononuclear cells (MNCs) were separated from CB and mPB samples (maximum after 1 day from harvesting) by stratification on Lympholyte-H 1.077 g/cm^3^ gradient (Gibco-Invitrogen, Milan, Italy), followed by red blood cell lysis for 15 min at 4°C. MNCs were then processed on magnetic columns for CD34^+^ cell isolation (mean purity 94 ± 4%) (CD34 Isolation kit; Miltenyi Biotec, Bologna, Italy), as previously described [25], and treated with our combination of cytokines on the same day. In selected cases, CD34^+^ cells from CB or mPB were cryopreserved in liquid nitrogen and then thawed before testing with the combined inflammatory cytokines. Of note, to minimize the influence of freezing/thawing, only thawed CD34^+^ cells with a survival rate > 80% were used and the thawed CB/mPB cells were studied in the same experiment.

### 2.3. Phenotype of Circulating CD34^+^ Cells

The phenotype of circulating CD34^+^ cells was evaluated in CB and mPB samples by conventional flow cytometry, as previously described [20]. Antibodies used to characterize the CD34^+^ cells are listed in Supplementary Table 1. A minimum of 1 × 10^4^ CD34^+^ cells were acquired by a BD Accuri C6 flow cytometer (Becton Dickinson, Milan, Italy). Analysis was performed excluding cellular debris in a SSC/FSC dot plot. The percentage of positive cells was calculated subtracting the value of the appropriate isotype controls. The absolute number of positive cells/μL was calculated as follows: percentage of positive cells × white blood cell count/100.

### 2.4. Apoptosis Assay

Freshly isolated CD34^+^ cells (2–5 × 10^5^) from CB units or mPB samples were maintained in RPMI 1640 with 10% fetal bovine serum (FBS), with or without IL-6 (10 ng/mL), IL-1*β* (1 ng/mL), TNF-*α* (10 ng/mL), and TIMP-1 (100 ng/mL), alone or in different combinations (all from Thermo Scientific, Rockford, IL, USA). After 24 hours, cells were stained for 15 min at RT with Annexin-V-FLUOS Staining Kit (Roche, Penzberg, Germany). Samples were then immediately analyzed by a BD Accuri C6 flow cytometer. Results are expressed as percentage of live cells compared to the whole cells.

### 2.5. Erythroid and Granulocytic Progenitor Assays

CB/mPB-derived CD34^+^ cells were cultured *in vitro* to achieve hematopoietic cell differentiation and the formation of colony-forming units (CFU-Cs), which is the sum of colony-forming unit-granulocyte macrophage (CFU-GM) and erythroid burst-forming units (BFU-E). Specifically, CD34^+^ cells were seeded in methylcellulose-based medium (human StemMACS HSC-CFU lite w/ Epo, Miltenyi Biotech) at 5 × 10^2^ cells/mL in 35 mm Petri dishes in the presence or absence of the selected proinflammatory factors: IL-6 (10 ng/mL), IL-1*β* (1 ng/mL), TNF-*α* (10 ng/mL), and TIMP-1 (100 ng/mL), alone or in combination. After 2 weeks of incubation at 37°C in 5% humidified CO_2_ atmosphere, CFU-C growth was evaluated by standard morphologic criteria using an inverted microscope (Axiovert 40, Zeiss, Milan, Italy).

### 2.6. Migration Assay

Migration of CB/mPB-purified CD34^+^ cells was assayed in transwell chambers (diameter 6.5 mm, pore size 8 *μ*m; Costar, Corning), as previously described [25]. In order to highlight the effects of the selected inflammatory cytokines, suboptimal CXCL12 gradient (120 ng/mL) was employed. Briefly, 50 *μ*L of RPMI 1640 plus 10% FBS containing 0.5 × 10^5^ cells was added to the upper chamber and 150 *μ*L of medium with or without CXCL12 ± IL-6 (10 ng/mL), IL-1*β* (1 ng/mL), TNF-*α* (10 ng/mL), and TIMP-1 (100 ng/mL) (alone or in combination) was added to the bottom chamber. After overnight incubation at 37°C in 5% humidified CO_2_ atmosphere, inserts (upper chambers) were removed and cells transmigrated into the lower chamber were recovered and counted by the Trypan Blue exclusion test. The amount of migrated cells was expressed as a percentage of the input, applying the following formula: (number of migrated cells recovered from the lower compartment/total number of cells loaded in the upper compartment) × 100. In addition, migrated cells were assayed in methylcellulose-based medium for their ability to form hematopoietic colonies (as above described).

### 2.7. Isolation and Expansion of Mesenchymal Stromal Cells (BMSCs) from Healthy Donors (HD)

BMSCs were obtained from BM aspirates collected from HD (*n* = 3), as previously described [26]. BM-MNCs were separated by stratification on Lympholyte-H 1.077 g/cm^3^ gradient (Gibco-Invitrogen) and then resuspended in culture medium (Dulbecco's modified Eagle's medium (DMEM) supplemented with 1% penicillin/streptomycin, L-glutamine, and 10% FBS), plated, and maintained in a humidified incubator at 37°C and 5% CO_2_. All nonadherent cells were removed after 24 hours. Medium was changed every 3–4 days until they reached 70–80% confluence. Cells were then trypsinized (Lonza, Verviers, Belgium), replated at a density of 3500 cells/cm^2^, and used for experiments within passages 3-4 after flow cytometry analysis for immunophenotype.

### 2.8. BMSCs Coculture Assay

In selected experiments, CB- and mPB-derived CD34^+^ cells were cocultured either without stromal support or directly seeded on a confluent layer of BMSCs in 96-well plates for 24 hours before use. CD34^+^ cells were then harvested and used to perform clonogenic and migration assay as above described; in addition, the selected cytokines were added to the bottom chamber. After overnight incubation at 37°C in 5% humidified CO_2_ atmosphere, cells transmigrated into the lower chamber were recovered and counted, as previously described.

### 2.9. Statistical Analysis

Data are presented as mean ± SEM of at least three independent determinations. Statistical differences between groups were determined by a two-tailed Student *t*-test and one- or two-way ANOVA, as appropriate. All analyses were performed using GraphPad Prism software (version 6.0; La Jolla, CA, USA); *p* ≤ 0.05 was considered to indicate statistical significance.

## 3. Results

### 3.1. Selected Combinations of Proinflammatory Cytokines Promote the In Vitro Survival of CB-Derived CD34^+^ Cells

To test the role of proinflammatory factors on HSPCs, we firstly evaluated the *in vitro* survival of CB- and mPB-derived CD34^+^ cells in the presence of IL-6, IL-1*β*, TNF-*α*, and TIMP-1, at concentrations previously shown by us to be effective in dose-response experiments [20].

**Figure 1:**
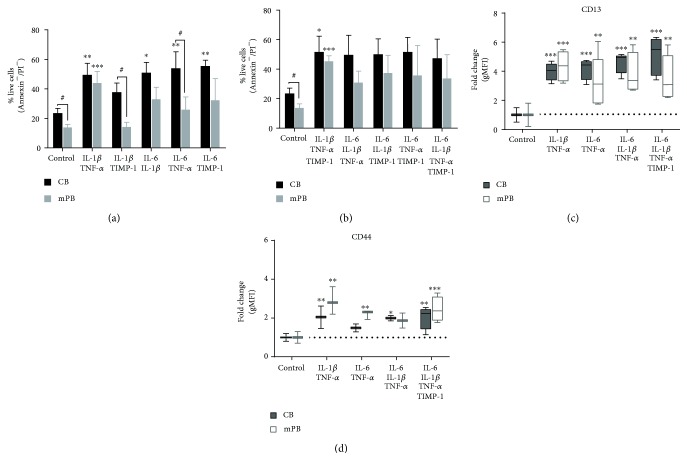
Survival and phenotype of CD34^+^ cells from CB or mPB in the presence of combined proinflammatory cytokines. (a) Percentage of live CD34^+^ cells from CB (indicated as negative for Annexin V and PI (black columns, *n* = 9) or mPB (grey columns, *n* = 8) *in vitro* treated for 24 hours with a two-by-two-factor combination and assessed using Annexin V/PI staining, as described in Methods. (b) Percentage of live CD34^+^ cells in the presence of multiple combinations of proinflammatory cytokines. (c–d) Box-plot graphs with fold change of gMFI for CD13 and CD44 expression in CD34^+^ cells after treatment with different combinations of inflammatory cytokines. Dot lines were used to mark control samples without any treatment. All data are presented as mean ± SEM of *n* (as above described) experiments performed in duplicate (^∗^ *p* ≤ 0.05, ^∗∗^ *p* ≤ 0.01, and ^∗∗∗^ *p* ≤ 0.001 versus untreated cells (control)). (^#^ *p* ≤ 0.05 CB versus mPB).

Spontaneous survival rate of CB-derived CD34^+^ cells was higher as compared to mPB counterparts (*p* ≤ 0.05; Figures 1(a) and 1(b)).

As shown in Figure S1, CB-derived CD34^+^ cell survival was further enhanced by TIMP-1, IL-1*β*, and IL-6 alone as compared with the mPB counterparts (*p* ≤ 0.05; resp.). Compared to untreated cells (control), TIMP-1, IL-1*β*, or IL-6 alone poorly promoted the survival of mPB- and CB-derived CD34^+^ cells with the notable exception of TNF-*α* which significantly (*p* ≤ 0.05) increased mPB-derived CD34^+^ cell survival.

Therefore, based on these results and on data reported in literature [20, 25, 27], we hypothesized that combinations of cytokines can make CB- or mPB-derived CD34^+^ cells more responsive to inflammatory stimuli. As shown in Figure 1(a), when cytokines were two-by-two combined, we found that IL-1*β* + TNF-*α* (*p* ≤ 0.01), IL-6 + IL-1*β* (*p* ≤ 0.05), or TNF-*α* (*p* ≤ 0.01) or TIMP-1 (*p* ≤ 0.01) significantly increased the percentage of viable CB-derived CD34^+^ cells as compared with the untreated counterparts. In contrast, only IL-1*β* + TNF-*α* significantly increased the survival rate of mPB-derived CD34^+^ cells (*p* ≤ 0.001).

Testing multiple cytokine combinations (Figure 1(b)), the survival of CB-derived and mPB-derived CD34^+^ cells was significantly increased in the presence of IL-1*β* + TNF-*α* + TIMP-1 (*p* ≤ 0.05 and *p* ≤ 0.001, resp.) as compared with untreated cells. When we compared CB and mPB (Figures 1(a) and 1(b)), the survival rate of CB-derived CD34^+^ cells was promoted in the presence of IL-1*β* + TIMP-1 and IL-6 + TNF-*α* (*p* ≤ 0.05).

These data suggest that CD34^+^ cells from CB are more actively responsive to inflammatory cues than their mPB counterparts; however, multiple combinations are required to promote their survival.

Subsequently, we examined whether a combination of proinflammatory factors would trigger CD34^+^ progenitor cell differentiation. In selected experiments, freshly isolated CD34^+^ cells were cultured in RPMI medium supplemented with or without additional proinflammatory factors for 24 hours. The expression of selected myeloid-specific markers (CD11c, CD13, CD14, and CD45) along with HSPC markers (CD38, CD133) or specific marker for cell adhesion/proliferation (CD44) was analyzed by flow cytometry. The expression of CD11c, CD14, CD38, CD45, and CD133 was not significantly affected by inflammatory factor treatment (data not shown). By contrast, after treatment with combined inflammatory cytokines, mPB- and CB-derived CD34^+^ cells upregulated the expression of CD13 and CD44 (Figures 1(c) and 1(d) and Supplementary Table 2). After treatment with IL-6 + IL-1*β* + TNF-*α* + TIMP-1, CB-derived CD34^+^ cells showed a 5-fold increase in geometric mean fluorescence intensity (gMFI) of CD13 as compared to untreated cells (gMFI, *p* ≤ 0.001). Accordingly, a statistically significant difference was also found in the presence of IL-1*β* + TNF-*α* or IL-6 + TNF-*α* ± IL-1*β* (4.41- and 4.64-fold change; *p* ≤ 0.001, resp.). A similar pattern was also found when mPB CD34^+^ cells were tested (Figure 1(c)).

Consistent with CD13 expression, the combination of IL-1*β* + TNF-*α* (*p* ≤ 0.01) and IL-6 + IL-1*β* + TNF-*α* (*p* ≤ 0.05) ± TIMP-1 (*p* ≤ 0.01) induced a significantly higher CD44 expression in CB-derived CD34^+^ cells (>2-fold increase, respectively; Figure 1(d)). When we evaluated the mPB-derived CD34^+^ cells, CD44 expression markedly increased in the presence of IL-1*β* + TNF-*α* (*p* ≤ 0.001), IL-6 + TNF-*α* (*p* ≤ 0.01), and the combination of cytokines altogether (*p* ≤ 0.001). Importantly, TNF-*α* alone increased the expression of CD13 and CD44 in CD34^+^ cells from both sources (Figure S2; *p* ≤ 0.001).

Taken together, these results demonstrate that selected combinations of inflammatory cytokines, along with the promotion of the survival of CB-derived CD34^+^ cells, stimulate the expression of CD13, which is an early and late myeloid marker.

### 3.2. Proinflammatory Cytokines Poorly Stimulate the Clonogenic Output of CD34^+^ Cells from CB and mPB

To confirm the capacity of selected proinflammatory cytokines to drive the HSPCs toward a myeloid lineage, we performed clonogenic assay.

**Figure 2:**
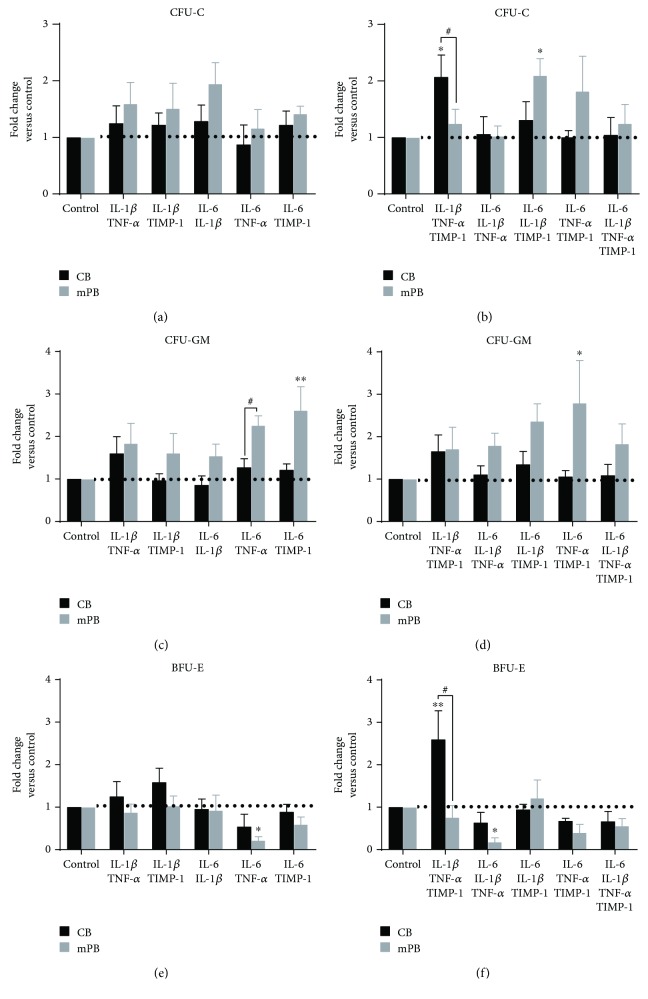
Clonogenic output of CB- and mPB-derived CD34^+^ cells after combined inflammatory stimuli treatment. Comparison of CFU-C formation between CB- (*n* = 9 independent experiments) and mPB-derived (*n* = 10 independent experiments) CD34^+^ cells cultured for 14 days in methylcellulose-based medium is shown. The total CFU-C output was assessed in the presence of inflammatory cytokines with the two-by-two combination (a) or multiple combinations (b). The CFU-GM (c–d) and BFU-E (e–f) output was assessed. The results are expressed as growth fold change of inflammatory cytokine-treated versus untreated cells (control). Control samples were marked with a dot line. All data are presented as mean ± SEM (^∗^ *p* ≤ 0.05, ^∗∗^ *p* ≤ 0.01 versus untreated cells (control)). (^#^ *p* ≤ 0.05 CB versus mPB).

We therefore evaluated the CFU-C growth in the presence of proinflammatory factors in a two-by-two combination. As shown in Figure 2(a) and Supplementary Table 3, CFU-C growth from CB or mPB-derived CD34^+^ cells was not significantly affected by incubation with the proinflammatory cytokines as compared with untreated cells. When we used multiple combinations of cytokines (Figure 2(b)), we found an increased clonogenic output in the presence of IL-1*β* + TNF-*α* + TIMP-1 for CB-derived CD34^+^ cells and in the presence of IL-6 + IL-1*β* + TIMP-1 for the mPB counterparts (*p* ≤ 0.05, resp.). Comparing the two sources, IL-1*β* + TNF-*α* + TIMP-1 led to an increase in the clonogenic output of CD34^+^ cells (*p* ≤ 0.05).

Of note, when colony composition was analyzed, no significant difference was observed in the CFU-GM growth (Figures 2(c) and 2(d)) between treated and untreated cells with the notable exception of IL-6 + TIMP-1 for mPB-derived CD34^+^ cells (*p* ≤ 0.01). Moreover, comparing the two sources, the CFU-GM growth of the mPB-derived CD34^+^ cells was significantly enhanced by IL-6 + TNF-*α* (*p* ≤ 0.05). As regards the erythroid lineage (Figures 2(e) and 2(f)), only IL-1*β* + TNF-*α* + TIMP-1 significantly promoted the BFU-E growth of CB CD34^+^ cells as compared to untreated cells and the mPB counterparts (*p* ≤ 0.01 and *p* ≤ 0.05, resp.). Interestingly, in the presence of IL-6 + TNF-*α* ± IL-1*β*, we found a statistically significant decrease in BFU-E growth of mPB CD34^+^ cells compared to untreated cells (*p* ≤ 0.05). Of note, IL-6 + TNF-*α* show opposite effects on the erythroid and granulomonocyte progenitors of mPB-derived CD34^+^ cells by enhancing CFU-GM (Figure 2(c)) and inhibiting BFU-E growth (Figure 2(e)).

To further investigate, we next examined the subtype compartment of CFU-GM and BFU-E as percentage of total CFU (Figure S3). As shown in Figure S3(A) and (B), we found that, after treatment with IL-6 + TNF-*α* ± IL-1*β* + TIMP-1, the CFU-GM growth of CB CD34^+^ cells was highly promoted (78.8 ± 8.4% and 74.5 ± 6.8% compared to 57% ± 3.2 of untreated cells; *p* ≤ 0.05, resp.), whereas it decreased in the presence of IL-1*β* + TNF-*α* + TIMP-1 (43.4 ± 3.7; *p* ≤ 0.05). No significant effects were observed when mPB CD34^+^ cells (Figure S3(C) and (D)) were analyzed.

These findings demonstrate that inflammatory cytokines slightly stimulate the hemopoietic functions of CB- and mPB-derived HSPCs.

### 3.3. Selected Combinations of Inflammatory Cytokines Mainly Enhance the In Vitro Migration of mPB-Derived CD34^+^ Cells

It has been reported that CXCL12 is chemotactic for CD34^+^ cells and that the migratory behavior of CD34^+^ cells depends on their source of origin [28].

**Figure 3:**
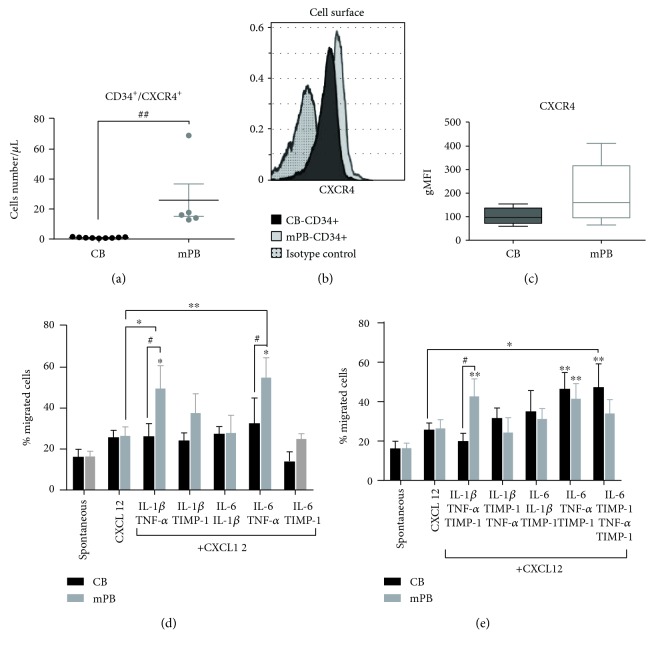
Migratory response of CB- and mPB-derived CD34^+^ cells in the presence of combined inflammatory stimuli. (a) The absolute number of circulating CD34^+^ cells from CB unit (*n* = 9) or mPB (*n* = 5) and coexpressing the CXCR4 receptor are shown. (b) Representative histogram of CXCR4 expression in CB- and mPB-derived CD34^+^ cells compared to isotype control. (c) Geometric mean value of CXCR4-positive cells on the CD34^+^ population after isolation from CB (*n* = 5) and mPB (*n* = 5) units. (d) Migration assay using transwell after o/n spontaneous migration (control) or exposure to CXCL12 (120 ng/mL) or to two-by-two inflammatory cytokines plus CXCL12 as chemoattractants. Percentages of migrated CD34^+^ cells from CB (black columns, *n* = 4) or mPB (grey columns, *n* = 4) are shown. (e) Migration assay using transwell after o/n spontaneous migration (control) or exposure to CXCL12 (120 ng/mL) or to CXCL12 plus multiple inflammatory cytokines as chemoattractants. Percentages of migrated CD34^+^ cells from CB (black columns, *n* = 4) or mPB (grey columns, *n* = 4) are shown. Data are presented as mean ± SEM of *n* (as above described) independent experiments (^∗^ *p* ≤ 0.05 and ^∗∗^ *p* ≤ 0.01 versus untreated cells (control) or CXCL12 alone) (^#^ *p* ≤ 0.05 and ^##^ *p* ≤ 0.01 CB versus mPB).

We firstly evaluated CXCR4 expression in CB- and mPB-CD34^+^ cells. As shown in Figure 3(a), the absolute number of CD34^+^ cells coexpressing CXCR4 was significantly increased in mPB as compared with the CB counterparts (*p* ≤ 0.01). Accordingly, we observed a slight increase in the geometric mean value of CXCR4 in mPB CD34^+^ cells as compared to CB-derived CD34^+^ cells (Figures 3(b) and 3(c)).

Subsequently, we compared spontaneous versus CXCL12-driven migration and no significant differences were found in the migration rate when CXCL12 was added in culture (Figures 3(d) and 3(e)). This was probably due to the fact that, to highlight the effects of the inflammatory cytokines, low CXCL12 dose (120 ng/mL) was used.

To study the role of proinflammatory cytokines in the modulation of spontaneous or CXCL12-mediated migration, we set up the *in vitro* migration of CB- or mPB-derived CD34^+^ cells in the presence of CXCL12 alone or CXCL12 plus selected combinations of proinflammatory cytokines (Figures 3(d) and 3(e)). As compared to the spontaneous migration, CD34^+^ cells from mPB showed increased migration ability toward CXCL12 when IL-1*β* + TNF-*α*, IL-6 + TNF-*α* (*p* ≤ 0.05, resp.), and IL-1*β* + TNF-*α* + TIMP-1 or IL-6 + TNF-*α* + TIMP-1 (*p* ≤ 0.01, resp.) were added. IL-6 + TNF-*α* + TIMP-1 ± IL-1*β* significantly increased the migration of the CB CD34^+^ cells (*p* ≤ 0.01, resp.). As compared to migration toward CXCL12 alone, only IL-1*β* + TNF-*α* or IL-6 + TNF-*α* significantly enhanced the migration of mPB-derived CD34^+^ cells (*p* ≤ 0.05 and *p* ≤ 0.01, resp.). The migratory capacity toward CXCL12 of CB CD34^+^ cells was promoted only by IL-6 + TNF-*α* + TIMP-1 + IL-1*β* (*p* ≤ 0.05). Comparing the two sources, the migration rate of CD34^+^ cells from mPB was significantly enhanced when CXCL12 + IL-1*β* + TNF-*α* ± TIMP-1 (*p* ≤ 0.05, resp.) and CXCL12 + IL-6 + TNF-*α* (*p* ≤ 0.05) were added to the lower transwell chamber.

These results demonstrate the capacity of selected combinations of inflammatory cytokines to increase the CXCR4-driven migration of CD34^+^ cells from mPB and CB. This effect was more prominent when mPB cells were assayed.

### 3.4. CD34^+^ Cells from mPB Show Increased Clonogenic Ability after In Vitro Migration toward Selected Combinations of Proinflammmatory Cytokines

Cell migration could be considered a selection of cells with different function and properties; for this reason, we tested the clonogenic potential of migrated CB and mPB CD34^+^ cells (Figure 2 and Supplementary Table 4).

**Figure 4:**
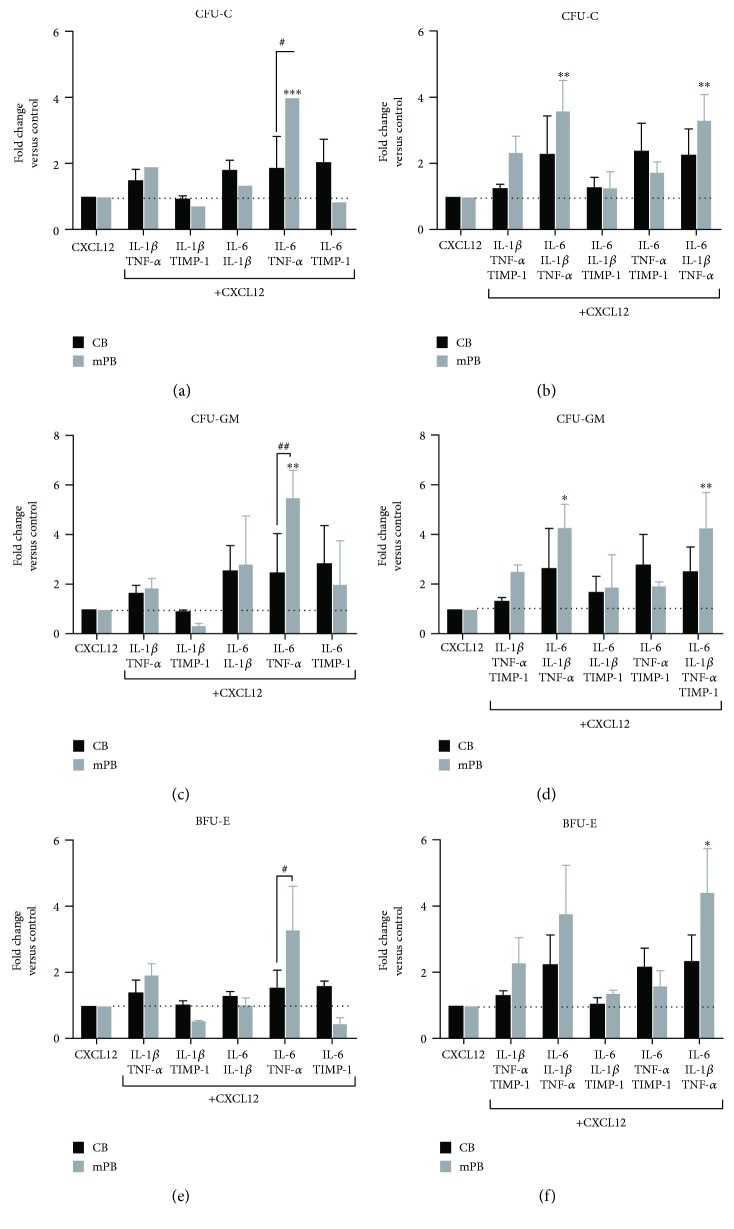
Clonogenic output of CB- or mPB-derived CD34^+^ cells after migration toward different combinations of inflammatory cytokines plus CXCL12. Panels a and b show the fold change of clonogenic potential of CB-derived (*n* = 4–8 independent experiments) and mPB-derived CD34^+^ cells (*n* = 4–9 independent experiments) after migration toward CXCL12 with or without the two-by-two combination (a) or multiple (b) combinations of proinflammatory factors (postmigration CFU-C). (c–d) Fold change of CFU-GM (c–d) and BFU-E (e–f) growth after migration towards CXCL12 in the presence of various combinations of cytokines. Dot lines were used to mark control samples after migration towards CXCL12. Results are expressed as mean fold change of CFU − C ± SEM (^∗^ *p* ≤ 0.05, ^∗∗^ *p* ≤ 0.01, and ^∗∗∗^ *p* ≤ 0.001; versus control cells (CXCL12)) (^#^ *p* ≤ 0.05 and ^##^ *p* ≤ 0.01 CB versus mPB).

Of note, at variance with the results of freshly isolated cells (Figure 2) in terms of clonogenic output, CXCL12 + IL-6 + TNF-*α* (*p* ≤ 0.001) ± IL1*β* (*p* ≤ 0.01) ± TIMP-1 (*p* ≤ 0.01) selected a subset of CD34^+^ cells from mPB with higher clonogenic potential as compared with CXCL12 alone (Figure 4(a). By contrast, no effects were found in the CB-derived counterparts (Figures 4(a) and 4(b)). Comparing the two sources, only the mPB-derived CD34^+^ cells migrated toward CXCL12 + IL-6 + TNF-*α* increased the CFU-C output (*p* ≤ 0.05) (Figures 4(a) and 4(b)). We then analyzed separately CFU-GM (Figures 4(c) and 4(d)) and BFU-E (Figures 4(e) and 4(f)) growth, observing a significant promotion of the CFU-GM growth in mPB CD34^+^ cells after migration toward CXCL12 + IL-6 + TNF-*α* and CXCL12 + IL-6 + IL-1*β* + TNF-*α* ± TIMP-1 as compared to CXCL12 alone (*p* ≤ 0.01, *p* ≤ 0.05 and *p* ≤ 0.01, resp.). Comparing the two sources, CFU-GM growth was higher after migration of mPB CD34^+^ cells toward CXCL12 + IL-6 + TNF-*α* (*p* ≤ 0.01) (Figure 4(c)). With regard the erythroid progenitors, no significant differences in BFU-E growth were observed between treated and untreated cells of either CB or mPB, with exception of the migrated mPB-derived CD34^+^ cells towards CXCL12 + all cytokines (*p* ≤ 0.05; Figures 4(e) and 4(f)). Comparing the two sources, mPB CD34^+^ cells showed higher clonogenic ability (*p* ≤ 0.05) after migration toward CXCL12 + IL-6 + TNF-*α*. When colony composition was analyzed (Figure S4), combined inflammatory factors do not significantly modify the CFU-GM/BFU-E proportion of migrated CD34^+^ cells of both sources.

Overall, here we demonstrate that selected combinations of proinflammatory cytokines promoted the CXCR4-driven migration of mPB-derived CD34^+^ cells with higher clonogenic ability and granulomonocytic potential.

### 3.5. The Copresence of BMSCs and Combined Inflammatory Cytokines Does Not Show Additive/Synergistic Effect in Terms of Hemopoietic Supportive Role

To mimic the in vivo niche and to investigate the role of normal BMSCs in the inflammation-driven functional behavior of normal HSPCs, we cocultured CB- or mPB-derived CD34^+^ cells with BMSCs from HD in the presence or absence of combined proinflammatory cytokines.

**Figure 5:**
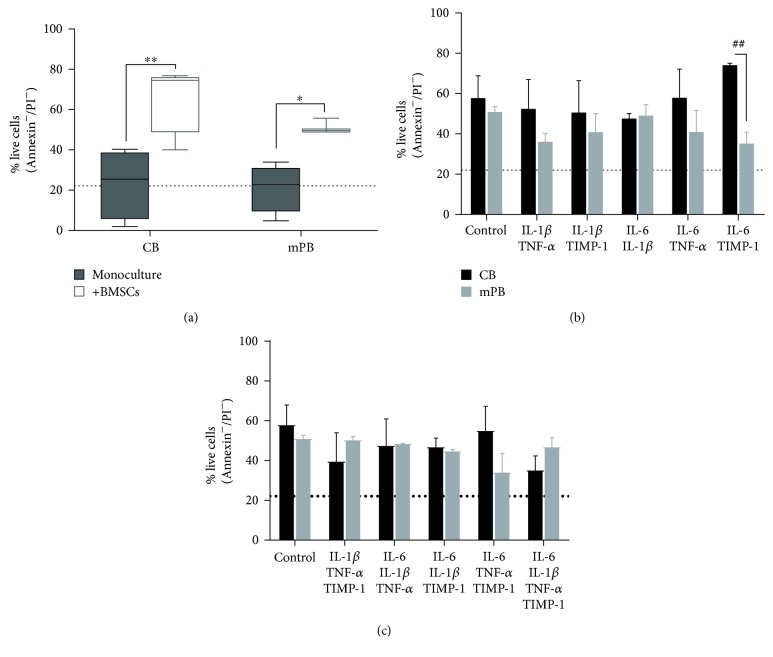
Survival of CB- and mPB-derived CD34^+^ cells after cocultures with HD-BMSCs and in the presence or the absence of combined inflammatory cytokines. (a) Comparison between 24-hour monocultures and cocultures of CB and mPB-derived CD34^+^ cells (*n* = 4 independent experiments, resp.) with BMSCs for *in vitro* survival (Annexin V/PI staining) is shown. Percentages of live CB- or mPB-CD34^+^ cells in the presence of BMSCs and/or proinflammatory cytokines with two by two combination (b) or multiple combinations (c) in comparison to CD34^+^ cells cocultured with BMSCs but without inflammatory stimuli are shown. For each graph, to highlight the comparison with cocultures, a dot line represented the mean percentage of live cells in all monocultures (CB and mPB CD34^+^ cells) (^∗^ *p* ≤ 0.05 and ^∗∗^ *p* ≤ 0.01 versus control cells) (^##^ *p* ≤ 0.01 CB vs mPB).

As shown in Figure 5(a), the survival of CD34^+^ cells, either from CB or mPB, was significantly promoted by BMSCs (*p* ≤ 0.01 and *p* ≤ 0.05, resp.). Interestingly, cocultures with BMSCs decreased the percentage of apoptotic CB CD34^+^ cells compared with the monoculture counterparts, with 33.5 ± 8.8% of apoptosis in cocultures versus 76.71 ± 8.9% in monocultures (*p* ≤ 0.05, data not shown). Similar results were obtained when cocultures of mPB CD34^+^ cells were performed, with 48.63 ± 2.1% of apoptosis in cocultures versus 78.92 ± 6.05% in monocultures (*p* ≤ 0.05) (data not shown). However, in the presence of BMSCs (Figures 5(b) and 5(c)), the viability of cocultured CD34^+^ cells from CB or mPB was not significantly modified by the combined inflammatory factors as compared with the untreated counterparts. Comparing the two sources, only IL-6 + TIMP-1 (*p* ≤ 0.01) significantly increased the number of viable cocultured CB-derived CD34^+^ cells.

Altogether, these findings demonstrate that (1) the survival of normal CD34^+^ cells is highly promoted by normal BMSCs through a strong protection from apoptosis, (2) BMSCs alone or the combined proinflammatory cytokines stimulate the survival of normal HSPCs at the same extent, and (3) the copresence of BMSCs and the combined inflammatory cytokines does not show additive/synergistic effect in terms of hemopoietic supportive role.

### 3.6. Proinflammatory Cytokines Do Not Modify the In Vitro Migration of CD34^+^ Cells from CB and mPB toward BMSCs

The BMSCs produce CXCL12 as mediator of migratory response of different cell types. CXCL12 is constitutively expressed by murine and human BM stromal cells [29]. To explore the effects of inflammation on the CXCR4-driven migratory ability of CD34^+^ cells in the presence or absence of BMSCs, we set up a migratory assay towards CXCL12 alone and BMSCs alone or in combination with various inflammatory cytokines. Of note, in order to mimic the in vivo pattern, along with inflammatory cytokines, a suboptimal concentration of CXCL12 (120 ng/mL) was also added.

**Figure 6:**
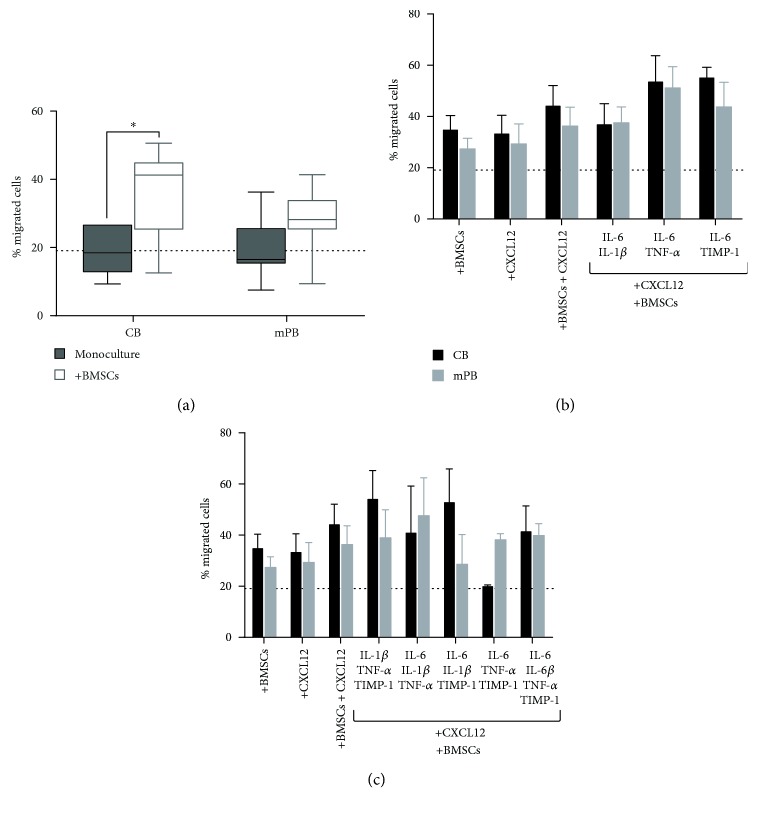
Migration of CB- and mPB-derived CD34^+^ cells towards CXCL12 and combined proinflammatory stimuli gradient and in the presence of normal BMSCs as further chemoattractant. (a) Comparison between spontaneous migration and migration toward BMSCs precultured on the bottom for 24 hours before seeding CD34^+^ cells on the top of the transwell system (*n* = 4 independent experiments, resp.). Percentages of migrated CD34^+^ cells (seeded on the top of transwell) towards BMSCs (seeded 24 hours before on the bottom) plus CXCL12 and proinflammatory cytokines (two-by-two combination (b) or multiple combinations (c)) as chemoattractants are shown. For each graph, to highlight the comparison with cocultures, a dot line represented the mean percentage of migrated cells towards CXCL12 in all monocultures (CB and mPB CD34^+^ cells) (^∗^ *p* ≤ 0.05 versus control cells).

As shown in Figure 6(a), we compared the spontaneous migration of CD34^+^ cells from CB and mPB with the migration towards BMSCs seeded on the bottom of the transwell system, as chemoattractant. We found that the migration of CD34^+^ cells from both sources was promoted by BMSCs, being significant (*p* ≤ 0.05) with CB only. However, the migration rate of CB or mPB CD34^+^ cells was not increased by the presence of CXCL12 + BMSCs as compared with that of CXCL12 alone or BMSCs alone (Figures 6(b) and 6(c)). When we added various combinations of proinflammatory cytokines in the presence of CXCL12 and BMSCs, once again we did not find any significant difference in the migration rate of CD34^+^ cells between treated and untreated cells or between the two sources (Figures 6(b) and 6(c)).

These experiments demonstrate that the BMSCs exert a potent chemoattractive effect on normal HSPCs; moreover, in the presence of BMSCs, these combined proinflammatory factors are unable to significantly modify the CXCR-4-driven migratory behavior of HSPCs from both sources.

## 4. Discussion

Several cytokine-based strategies enhancing hemopoiesis, homing, and subsequent engraftment of CB/mPB-derived HSCs have been previously described [30]. However, critical steps are involved in these processes and further insights are necessary to better understand HSPCs homing and engraftment [31]. Along with a role as activators of immune cell function, a growing evidence now demonstrates that proinflammatory cytokines strongly affect the size and lineage distribution of the blood cells via reprogramming of HSC/HSPC and the supporting BM niche [5, 32]. Along with the cytokine storm, the network created by danger-associated molecular patterns (PAMPs/DAMPs) and alarmins could deviate HSCs fate, directly or indirectly via stromal cells [33]. Based on these evidences and due to the lack of informative data, it is of utmost importance to clarify the impact of proinflammatory cytokines on the biology of the normal HSPC and its BM microenvironment. A better understanding of the mechanisms driven by the inflammatory milieu in HSPCs may lead to better transplantation outcomes and knowledge of hematological defects or malignancies.

Here we tested various combinations of proinflammatory cytokines such as IL-1*β*, IL-6, TNF-*α*, and TIMP-1 in order to investigate their functional role on the *in vitro* behavior of young (CB-derived) and adult (mPB-derived) CD34^+^ cells. To mirror the *in vivo* condition, tested cytokines were two to two or multiple combined. We selected these inflammatory cytokines for the following features: (i) IL-6 is a pleiotropic proinflammatory cytokine that acts on many cell types including hemopoietic cells. It has been implicated as a critical activator of myelopoiesis in response to pathogen infection and chronic inflammation [34]; (ii) IL-1*β* is a potent inflammatory cytokine that mediates leukocytosis and thrombocytosis under inflammatory conditions by inducing various cytokines (i.e., granulocyte colony-stimulating factor and IL-6) [7]. Moreover, Pietras et al. [35] recently demonstrated that while IL-1*β* is dispensable for steady-state hemopoiesis, acute exposure to IL-1*β* accelerates HSC proliferation and instructs HSC priming for a myeloid fate. Lastly, it is involved in the pathogenesis of solid tumors and hematological malignancies [36]; (iii) TNF-*α* negatively regulates the expansion and self-renewal of HSPCs [37]. However, other evidence suggests that TNF signalling may enhance HSC function [38, 39]; (iiii) TIMP-1, through receptor (CD63) binding, has a role in multiple biological processes, including inflammation and immune regulation. We recently demonstrated that it displays cytokine-like features in the normal and leukemic HSPC compartment [25, 26].

In addition, experimental evidence demonstrated that combined proinflammatory cytokines such as IL-6 and TNF-*α* are critical for both inflammation and cancer by activating STAT3 and the NF-*κ*B complex [40]. Furthermore, other inflammatory cytokines and pathways (such as IL-6) are induced by IL-1*β* and are involved in malignancy [36]. Thus, the combined action of these cytokines could constitute a central signalling pathway that promotes inflammation and tumor growth.

Comparing the two sources (neonatal versus adult) of HSPCs, here we demonstrated that the following:

1. Various combinations of inflammatory cytokines mainly enhance the *in vitro* survival of CB-derived CD34^+^ cells.
2. As compared to CB, mPB-derived CD34^+^ cells are susceptible to selected combinations of inflammatory factors in terms of proliferation and hemopoietic function.
3. TNF-*α*, alone or in combination, promotes the expression of the CD13 myeloid marker and CD44, an adhesion/proliferation marker of normal and leukemic cells.
4. Along with an increased number of circulating CXCR4^+^CD34^+^ cells, selected combinations of inflammatory cytokines mainly enhance the *in vitro* migration of mPB-derived CD34^+^ cells.
5. BMSCs alone or combined inflammatory cytokines promote survival/migration of HSPCs from both sources at the same extent; moreover, their copresence does not show additive/synergistic effect in terms of hemopoietic supportive role.

Of note, despite that the use of frozen/thawed CD34^+^ cells in some cases might have influenced the variability of phenotype/clonogenic ability, our results clearly demonstrate that the selected network of proinflammatory factors has the potential to activate either neonatal or adult normal hemopoiesis and acts as regulator of HSPCs. Moreover, these results are consistent with previously described promoting effects of the inflammatory microenvironment on hemopoiesis [4,7,12,]. Taking into account that the functional consequences of inflammation-related molecules depend on the duration of exposure (acute versus chronic), our results may provide a starting point to investigate whether the inflammatory cues contribute to creating a favorable milieu for the development of hematological malignancies through hemopoietic activation.

Several groups evaluated the expression of hematopoietic markers, identifying various subpopulations of CD34^+^ cells in CB or mPB samples [41, 42]. Interestingly, we found that, when CB- or mPB-derived CD34^+^ cells were treated with combined proinflammatory cytokines including TNF-*α*, CD13 expression was highly promoted. The CD13/aminopeptidase N is expressed on various cell types, including myeloid hematopoietic cells, and regulates biological phenomena such as differentiation, proliferation, apoptosis, motility, and tumor cell invasion. Importantly, CD13 degrades the chemokine CXCL11 and modulates CXCL12-induced migration [43, 44]. It has also been reported that high levels of human CD13 correlate with leukemic cell resistance to apoptosis [45]. Moreover, CD13 is differentially expressed in discrete states of differentiation of neoplastic myeloid cells [46]. Along with CD13, here we found modulation of the homing-associated cell adhesion molecule CD44. Recently, CD44, as a receptor for hyaluronan, emerges as mediator of cell-cell and cell-matrix interactions and as pivotal trigger in cancer stem cell communication with their microenvironment [47, 48]. Here we identified combined proinflammatory cytokines including TNF-*α*, which are able to upregulate CD44 expression on the surface of normal CD34^+^ cells. Although TNF-*α*-driven modulation of CD44 expression was already reported in several cancers [49], this is the first time that a strong link has been found between combined inflammatory cytokines and CD44 expression on HSPCs. Therefore, our results clearly indicate that the inflammation-driven CD13 and CD44 upregulation on neonatal or adult CD34^+^ cells has the potential of modulating key functional pathways (i.e., survival/differentiation) of the normal hemopoietic progenitor cells. Interestingly, these pathways may also play a role in myeloproliferation and leukemogenesis.

Several studies have shown that HSC can be expanded in cytokine-driven culture and by MSC feed layers [50, 51]. Consistently, our data clearly showed that normal BMSCs enhance survival and migration of CB- and mPB-derived CD34^+^ cells. Due to their capacity to modulate oxidative stress, it is likely that BMSCs are capable of inhibiting apoptosis; moreover, producing CXCL12 [52], they enhance cell migration. Interestingly, even though it is likely that different mechanisms are involved, the BMSC-driven promoting effect of the CB-derived CD34^+^ cell survival is similar to that induced by the combined inflammatory cytokines. A similar trend was observed in mPB. However, for the first time, we investigated the copresence of BMSCs and various combinations of selected proinflammatory cytokines. Surprisingly, the copresence of inflammatory stimuli with BMSCs did not significantly modify the survival-migration rate of normal HSPCs as compared with that observed after stimulation with BMSCs alone or combined inflammatory cytokines alone. Of note, the BMSCs are capable to sustain the survival of mPB-derived CD34^+^ cells in the presence of IL-1*β* + TIMP-1. These findings demonstrate that in our culture system an acute inflammatory stimulus does not impair the hemopoietic-supportive role of BMSCs. Interestingly, based on murine models, it has been previously demonstrated that MSCs can modulate inflammation by secreting soluble receptors for IL-1 and TNF, which bind to IL-1 and TNF-*α* and neutralize the activity of the cytokines [53–55]. We can therefore hypothesize that in our cocultures the promotion of normal HSPCs survival/migration is mainly due to BMSCs which show regulatory properties of the hemopoietic function of HSPCs because they are capable of balancing the proinflammatory signal-driven hemopoietic activation. These results suggest that exploiting or modulating the thin balance between pro- and anti-inflammatory pathways may be a clinically relevant approach in hematological malignancies.

## 5. Conclusion

The goal of this study is the demonstration that an inflammatory microenvironment promotes distinct *in vitro* functional activation of neonatal and adult HSPCs and that an acute inflammatory stress does not impair the hemopoietic promoting effect of BMSCs. Moreover, this study may represent a starting point for future studies aiming at addressing the role of inflammation and the balance with anti-inflammatory signals in the functional behavior of normal HSPCs and their transformation to a leukemic phenotype.

## Data Availability

The data used to support the findings of this study are available from the corresponding author upon request.

## Supplementary Materials

Supplementary Materials Supplementary Figure 1: survival of CD34^+^ cells derived from CB or mPB in the presence of 517 proinflammatory cytokines alone. Supplementary Figure 2: phenotype of CD34^+^ cells derived from CB or mPB in the presence of proinflammatory cytokines alone. Supplementary Figure 3: colony composition in the presence or absence of 521 combined inflammatory cytokines. Supplementary Figure 4: colony composition of CB- and mPB-derived CD34^+^ cells after migration toward CXCL12 alone and CXCL12 plus various combinations of inflammatory factors. Supplementary Table 1: antibodies used to characterize the CD34^+^ cells. Supplementary Table 2: absolute numbers of gMFI for CD13 and CD44 expression in CD34^+^ cells from CB or mPB after incubation for 24 hours in the presence/absence of 528 inflammatory stimuli. Supplementary Table 3: absolute numbers of CFU-C, CFU-GM, and BFU-E after incubation of CD34^+^ cells from CB or mPB in methylcellulose-based medium for 14 days in the presence/absence of inflammatory stimuli. Supplementary Table 4: absolute numbers of CFU-C, GM-CFU, and BFU-E in CD34^+^ derived from CB or mPB counted after migration towards inflammatory stimuli and seeded in methylcellulose-based medium for 14 days.
