# Supplementary material for: Mobilized Peripheral Blood versus Cord Blood: Insight into the Distinct Role of Proinflammatory Cytokines on Survival, Clonogenic Ability, and Migration of CD34+ Cells
Source: Mediators Inflamm. 2018 Jul 4;2018:5974613. doi: 10.1155/2018/5974613 (PMC6079419; doi:10.1155/2018/5974613)
Supplement: Supplementary Materials — Supplementary Figure 1: survival of CD34+ cells derived from CB or mPB in the presence of 517 proinflammatory cytokines alone. Supplementary Figure 2: phenotype of CD34+ cells derived from CB or mPB in the presence of proinflammatory cytokines alone. Supplementary Figure 3: colony composition in the presence or absence of 521 combined inflammatory cytokines. Supplementary Figure 4: colony composition of CB- and mPB-derived CD34+ cells after migration toward CXCL12 alone and CXCL12 plus various combinations of inflammatory factors. Supplementary Table 1: antibodies used to characterize the CD34+ cells. Supplementary Table 2: absolute numbers of gMFI for CD13 and CD44 expression in CD34+ cells from CB or mPB after incubation for 24 hours in the presence/absence of 528 inflammatory stimuli. Supplementary Table 3: absolute numbers of CFU-C, CFU-GM, and BFU-E after incubation of CD34+ cells from CB or mPB in methylcellulose-based medium for 14 days in the presence/absence of inflammatory stimuli. Supplementary Table 4: absolute numbers of CFU-C, GM-CFU, and BFU-E in CD34+ derived from CB or mPB counted after migration towards inflammatory stimuli and seeded in methylcellulose-based medium for 14 days. [file 5974613.f1.pdf]

Supplementary Material

Supplementary Figure 1. Survival of CD34$^+$ cells derived from CB or mPB in the presence of pro-inflammatory cytokines alone. Percentages of live CD34$^+$ cells from CB (black columns, *n* = 9) or mPB (grey columns, *n* = 8) after *in vitro* treatment for 24 hours with inflammatory cytokines alone (Annexin V/PI staining) are shown. All data are presented as mean ± SEM. (**p ≤ 0.05; vs untreated cells (Control)*) (*#p ≤ 0.05;CB vs mPB)*.

Supplementary Figure 2. Phenotype of CD34$^+$ cells derived from CB or mPB in the presence of pro-inflammatory cytokines alone. Box-plot graphs with fold-change of gMFI of CD13 and CD44 expression in CD34$^+$ cells after treatments with the inflammatory cytokines alone are shown. Dot lines were used to mark Control samples without any treatment. All data are presented as mean ± SEM. (****p ≤ 0.001 vs untreated cells (Control)*).

Supplementary Figure 3. Colony composition in the presence or the absence of combined inflammatory cytokines. A) Bar-plot with percentages of the different subtypes of BFU-E and CFU-GM in CB CD34$^+$ cells as compared to the total number of CFU-C after 14 days in methylcellulose-based medium, in the presence of the two-by-two cytokines combination (A) or multiple combinations (B). Bar-plot with percentages of BFU-E and CFU-GM as referred to mPB-derived CD34$^+$ cells are shown in panels C-D. All data are presented as mean ± SEM. (**p ≤ 0.05 vs untreated cells (Control)*)

Supplementary Figure 4. Colony composition of CB- and mPB derived CD34$^+$ cells after migration toward CXCL12 alone and CXCL12 plus various combinations of inflammatory factors. Bar-plots with the percentages of BFU-E and CFU-GM growth as compared with the total number of CFU-C after 14 days in methylcellulose-based medium for CB CD34$^+$ cells after migration towards CXCL12 ± combined pro-inflammatory cytokines are shown (A,B). Bar-plots

of mPB-derived CD34^{+} cells are shown in panels C and D. All data are presented as mean ± SEM. No significant differences were observed.

Supplementary Table 1

The following Monoclonal Antibodies were used to phenotypically characterize the mPB- and CB-derived CD34^{+} cells:

- anti-human CD11c (clone S-HCL-3)
- anti-human CD13 (clone WM15)
- anti-human CD14 (clone HCD14)
- anti-human CD34 (clone 8G12)
- anti-human CD38 (clone HIT2)
- anti-human CD44 (clone G44-26)
- anti-human CD45 (clone HI30)
- anti-human CD133 (clone W6B3C1)
- anti-human CD184 (CXCR4; clone 12G5)

All from BD Biosciences (San Jose, CA USA)

Negative controls were isotype-matched irrelevant MoAbs (BD Biosciences and Miltenyi Biotech).

Lucia Catani, Supplementary 1, top

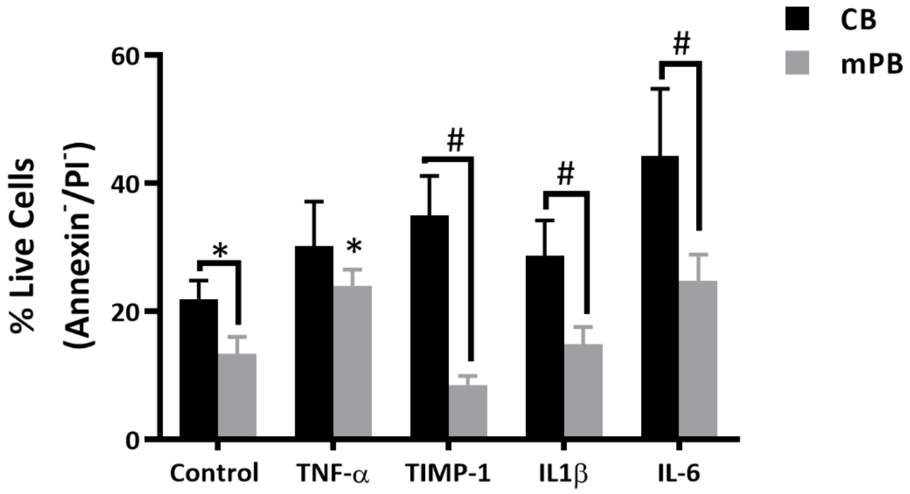

Lucia Catani, Supplementary Figure 2, top

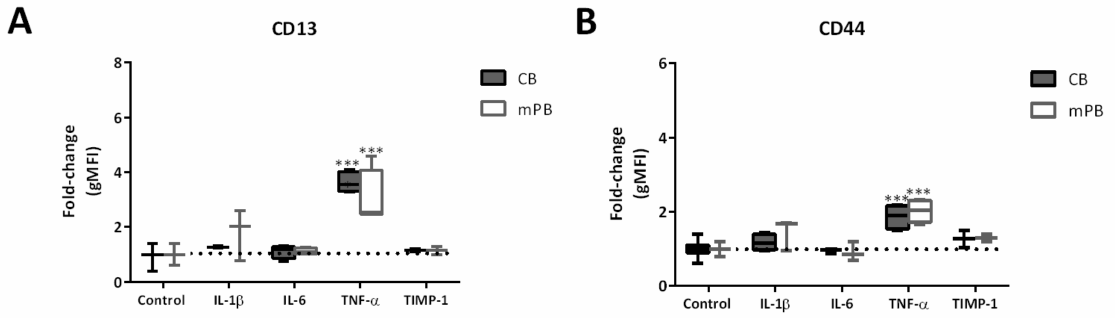

Lucia Catani, Supplementary Figure 3, top

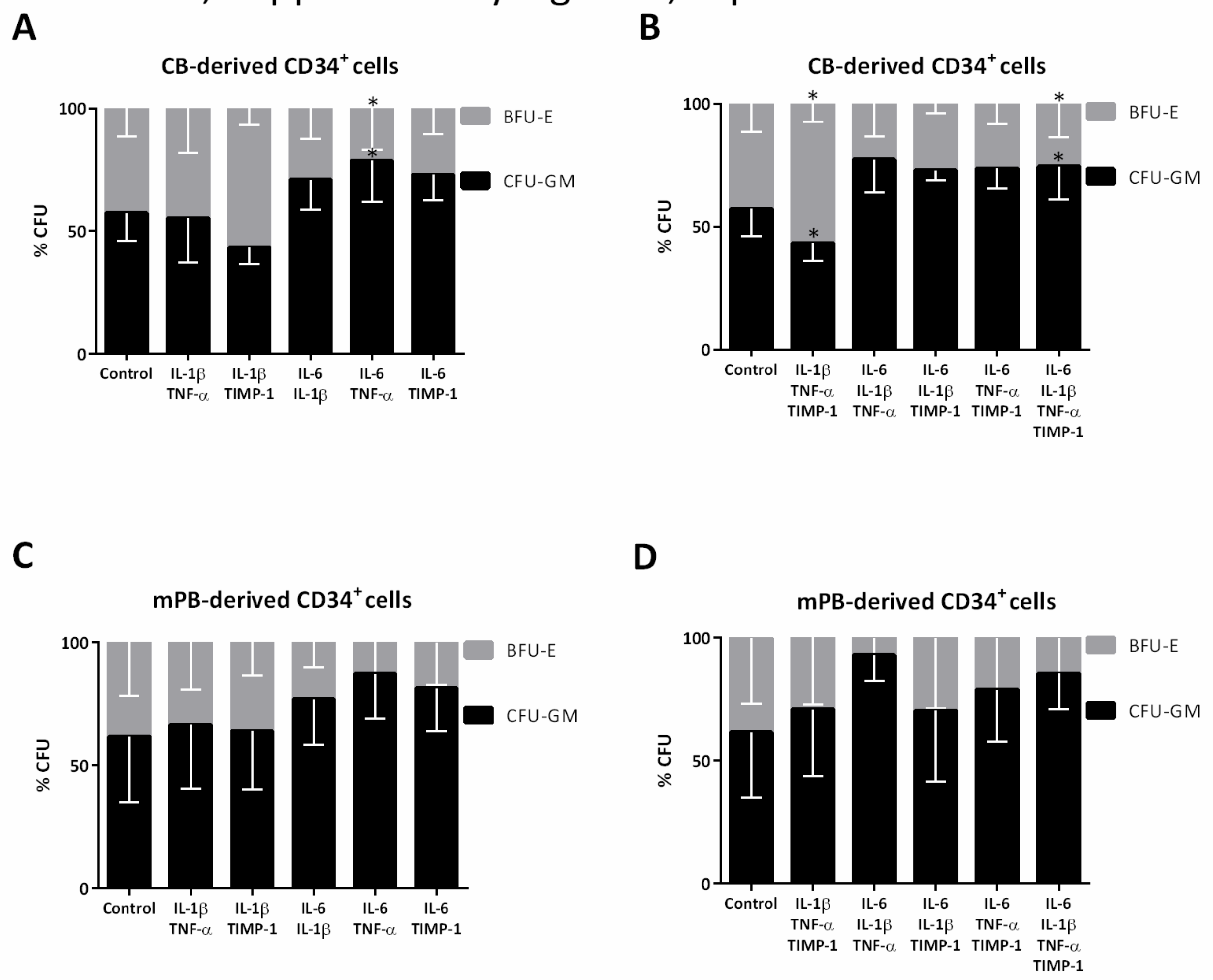

Lucia Catani, Supplementary Figure 4, top

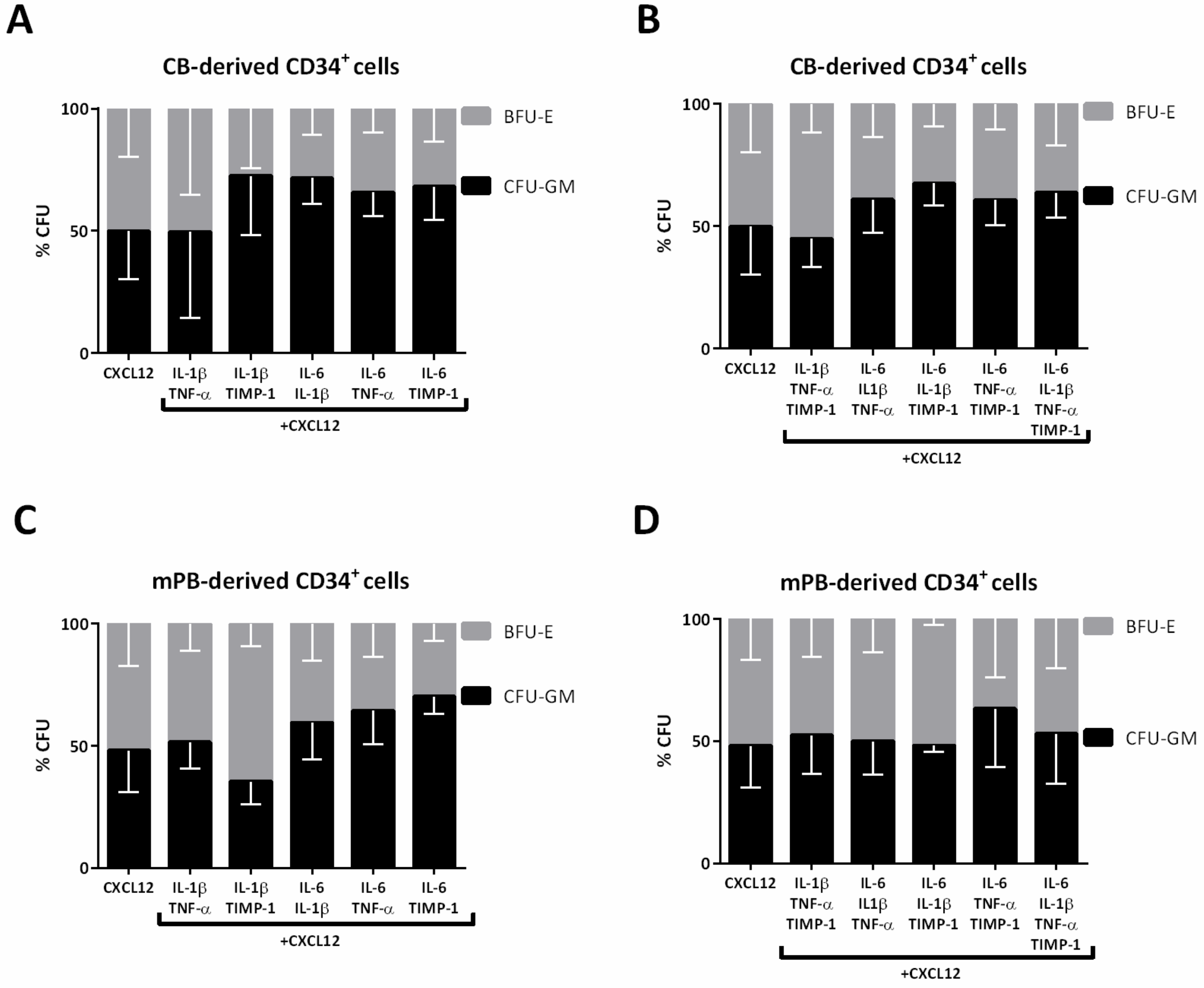

Supplementary Table 2. Absolute numbers of gMFI for CD13 and CD44 expression in CD34^{+} cells from CB or mPB after incubation for 24 hours in the presence/absence of inflammatory stimuli.

Values represent the means ± Standard Error from 4-6 independent experiments. Control values for each treatment is shown in brackets.

Treatment	Source	CD13 gMFI
IL-1β	CB	411 ± 15 (639 ± 106)
	mPB	560 ± 96.8 (454 ± 249)
TNF-α	CB	1677 ± 398 (639 ± 106)
	mPB	2206 ± 492 (933 ± 216)
IL6	CB	733 ± 108 (639 ± 106)
	mPB	1088 ± 372 (933 ± 216)
IL-1β TNF-α	CB	2278 ± 448 (639 ± 106)
	mPB	856 ± 25.66 (206 ± 42)
IL6 IL-1β	CB	1028 ± 146 (639 ± 106)
	mPB	1362 ± 357 (933 ± 216)
IL6 TNF-α	CB	3049 ± 572 (639 ± 106)
	mPB	3702 ± 289 (933 ± 216)
IL6 IL-1β TNF-α	CB	3346 ± 541 (639 ± 106)
	mPB	4169 ± 526 (933 ± 216)
IL6 IL-1β TNF-α TIMP-1	CB	3157 ± 950 (639 ± 106)
	mPB	2768 ± 658 (933 ± 216)

Treatment	Source	CD44<br>gMFI
**IL-1β**	**CB**	2942 ± 275<br>(2506 ± 23)
	**mPB**	4739 ± 1401<br>(5596 ± 1164)
**TNF-α**	**CB**	8642 ± 2056<br>(4938 ± 1432)
	**mPB**	13074 ± 2734<br>(5596 ± 1164)
**IL6**	**CB**	6806 ± 543<br>(4938 ± 1432)
	**mPB**	5782 ± 2257<br>(5596 ± 1164)
**IL-1β**<br>**TNF-α**	**CB**	10934 ± 2932<br>(4938 ± 1432)
	**mPB**	5222 ± 388<br>(2005 ± 374)
**IL6**<br>**IL-1β**	**CB**	9159 ± 2145<br>(4938 ± 1432)
	**mPB**	7191 ± 1320<br>(5596 ± 1164)
**IL6**<br>**TNF-α**	**CB**	11090 ± 2518<br>(4938 ± 1432)
	**mPB**	17199 ± 1963<br>(5596 ± 1164)
**IL6**<br>**IL-1β**<br>**TNF-α**	**CB**	14775 ± 2371<br>(4938 ± 1432)
	**mPB**	12695 ± 2847<br>(5596 ± 1164)
**IL6**<br>**IL-1β**<br>**TNF-α**<br>**TIMP-1**	**CB**	8138 ± 3245<br>(4938 ± 1432)
	**mPB**	9945 ± 2393<br>(5596 ± 1164)

Supplementary Table 3. Absolute numbers of CFU-C, CFU-GM and BFU-E after incubation of CD34$^+$ cells from CB or mPB in methylcellulose-based medium for 14 days in the presence/absence of inflammatory stimuli

Values represent the means ± Standard Error from 4-12 independent experiments.

Treatment	Source	Colonies<br>CFU-C	GM-CFU	BFU-E
IL-1β<br>TNF-α	CB	74.6 ± 28.07<br>(67.9 ± 17.19)	39.8 ± 17.11<br>(27.4 ± 9.92)	34.8 ± 12.17<br>(30.5 ± 7.99)
	mPB	63.38 ±17.02<br>(51.63 ± 13.08)	37 ± 8.75<br>(29 ± 7.99)	26.38 ± 9.44<br>(22.75 ± 6.44)
IL-1β<br>TIMP-1	CB	70.83 ± 18.81<br>(67.9 ± 17.19)	30.83 ± 9.009<br>(44.08 ± 15.59)	40 ± 10.54<br>(30.5 ± 7.99)
	mPB	56.71 ± 21.58<br>(46.14 ± 13.71)	30.29 ± 9.68<br>(26.43 ± 8.74)	26.43 ± 12.42<br>(22.75 ± 6.44)
IL6<br>IL-1β	CB	153.3 ± 17.34<br>(123.2 ± 14.64)	106.3 ± 8.509<br>(88 ± 11.11)	47 ± 13.07<br>(41.25 ± 6.36)
	mPB	51.4 ± 20.68<br>(28 ± 11.26)	39.6 ± 18.2<br>(18.6 ± 8.99)	11.8 ± 5.59<br>(9.4 ± 4.67)
IL6<br>TNF-α	CB	96 ± 26.98<br>(123.2 ± 14.64)	69.25 ± 12.07<br>(88 ± 11.11)	26.75 ± 17.21<br>(41.25 ± 6.37)
	mPB	30.5 ± 10.53<br>(28 ± 11.26)	26.5 ± 10.16<br>(18.6 ± 8.99)	4 ± 1.673<br>(12.33 ± 4.81)
IL6<br>TIMP-1	CB	142.8 ± 17.88<br>(123.2 ± 14.64)	102.3 ± 10.4<br>(88 ± 11.11)	40.5 ± 11.59<br>(41.25 ± 6.369)
	mPB	38.75 ± 11.1<br>(28 ± 11.26)	29.25 ± 9.264<br>(15.7 ± 6.68)	9.5 ± 3.16<br>(12.33 ± 4.81)

Treatment	Source	Colonies<br>CFU-C	GM-CFU	BFU-E
IL-1β<br>TNF-α<br>TIMP-1	CB	143.3 ± 19.44<br>(79 ± 14.07)	64 ± 13.62<br>(43.25 ± 10.02)	79.25 ± 7.28<br>(35.75 ± 7.16)
	mPB	52.29 ± 14.74<br>(46.14 ± 13.71)	32.72 ± 10.67<br>(26.43 ± 8.74)	19.57 ± 7.43<br>(19.71 ± 6.66)
IL6<br>IL-1β<br>TNF-α	CB	119.4 ± 23.09<br>(123.2 ± 14.64)	89.4 ± 9.71<br>(85 ± 9.11)	30 ± 14.41<br>(35.75 ± 7.15)
	mPB	28.2 ± 10.68<br>(28 ± 11.26)	25.6 ± 10.08<br>(18.6 ± 8.99)	2.6 ± 1.53<br>(9.4 ± 4.67)
IL6<br>IL-1β<br>TIMP-1	CB	151.3 ± 22.21<br>(123.2 ± 14.64)	110.3 ± 15.63<br>(85 ± 9.11)	41 ± 7.51<br>(35.75 ± 7.15)
	mPB	46.5 ± 19.17<br>(28 ± 11.26)	36.67 ± 16.9<br>(20.45 ± 6.21)	9.83 ± 5.77<br>(9.4 ± 4.67)
IL6<br>TNF-α<br>TIMP-1	CB	114.3 ± 9.40<br>(123.2 ± 14.64)	83.67 ± 2.96<br>(85 ± 9.11)	30.67 ± 7.83<br>(41.25 ± 6.36)
	mPB	25 ± 7.67<br>(28 ± 11.26)	17.8 ± 5.01<br>(11.08 ± 4.51)	7.2 ± 3.37<br>(9.4 ± 4.67)
IL6<br>IL-1β<br>TNF-α<br>TIMP-1	CB	120 ± 18.95<br>(129.3 ± 17.21)	87.5 ± 11.59<br>(85 ± 9.11)	32.5 ± 13.17<br>(41.25 ± 6.37)
	mPB	38.4 ± 19.98<br>(28 ± 11.26)	33.6 ± 19.43<br>(20.45 ± 6.21)	4.8 ± 2.01<br>(9.4 ± 4.67)

Supplementary Table 4. Absolute numbers of CFU-C, GM-CFU and BFU-E in $CD34^{+}$ derived from CB or mPB counted after migration towards inflammatory stimuli and seeded in methylcellulose-based medium for 14 days.

Values represent the means ± Standard Error from 4-6 independent experiments. Control values for each treatments in brackets.

Treatment	Source	Colonies<br>CFU-C	GM-CFU	BFU-E
IL-1β<br>TNF-α	CB	101.7 ± 21.94<br>(77.67 ± 23.62)	36.33 ± 15.6<br>(21 ± 6.08)	65.33 ± 8.686<br>(56.67 ± 19.38)
	mPB	61.75 ± 26.03<br>(30 ± 8.88)	28.25 ± 10.7<br>(14 ± 4.49)	33.5 ± 15.57<br>(16 ± 4.7)
IL-1β<br>TIMP-1	CB	75.63 ± 15.02<br>(77.67 ± 23.62)	42.3 ± 12.63<br>(44.83 ± 12.64)	33.33 ± 2.404<br>(48 ± 20.51)
	mPB	17 ± 3.215<br>(30 ± 8.88)	6.333 ± 2.028<br>(14 ± 4.49)	10.67 ± 1.333<br>(16 ± 4.7)
IL6<br>IL-1β	CB	126 ± 33.62<br>(99.55 ± 31.51)	93 ± 31.76<br>(52.33 ± 25.89)	33 ± 7.234<br>(30 ± 5.43)
	mPB	28.67 ± 7.839<br>(20.5 ± 6.58)	15.67 ± 6.84<br>(8 ± 3.51)	13 ± 5<br>(8 ± 4)
IL6<br>TNF-α	CB	123.7 ± 33.51<br>(99.55 ± 31.51)	81 ± 23.59<br>(52.33 ± 25.89)	42.67 ± 12.12<br>(30 ± 5.43)
	mPB	69 ± 12.26<br>(20.5 ± 6.58)	41.6 ± 4.13<br>(8 ± 3.51)	27.4 ± 9.532<br>(8 ± 4)
IL6<br>TIMP-1	CB	128.5 ± 46.5<br>(99.55 ± 31.51)	92 ± 44<br>(52.33 ± 25.89)	36.5 ± 2.5<br>(30 ± 5.43)
	mPB	13.5 ± 9.5<br>(15.5 ± 0.5)	9 ± 6<br>(8 ± 3.51)	4.5 ± 3.5<br>(8 ± 4)

Treatment	Source	Colonies<br>CFU-C	GM-CFU	BFU-E
IL-1β<br>TNF-α<br>TIMP-1	**CB**	92.2 ± 13.27<br>(74.2 ± 10.09)	39 ± 6.34<br>(34.2 ± 9.87)	53.2 ± 11.2<br>(40 ± 5.1)
	**mPB**	72.25 ± 23.93<br>(30 ± 8.88)	32.5 ± 8.836<br>(14 ± 4.49)	39.75 ± 15.21<br>(16 ± 4.70)
IL6<br>IL-1β<br>TNF-α	**CB**	142 ± 24.11<br>(71.67 ± 20.88)	82.4 ± 11.58<br>(56.17 ± 18.16)	59.6 ± 16.61<br>(25.33 ± 4.87)
	**mPB**	77.33 ± 13.02<br>(22 ± 8.07)	38.67 ± 8.452<br>(9 ± 3.13)	38.67 ± 9.735<br>(13 ± 5.58)
IL6<br>IL-1β<br>TIMP-1	**CB**	83.33 ± 15.84<br>(71.67 ± 20.88)	56.67 ± 12.25<br>(56.17 ± 18.16)	26.67 ± 6.692<br>(25.33 ± 4.87)
	**mPB**	20 ± 8<br>(22 ± 8.07)	9.5 ± 3.5<br>(6.33 ± 2.33)	10.5 ± 4.5<br>(8 ± 4)
IL6<br>TNF-α<br>TIMP-1	**CB**	112 ± 15.69<br>(71.67 ± 20.88)	66.25 ± 7.521<br>(56.17 ± 18.16)	45.75 ± 10.83<br>(21.4 ± 3.53)
	**mPB**	36.2 ± 3.007<br>(19 ± 3.27)	23.6 ± 4.986<br>(13.2 ± 2.81)	12.6 ± 3.076<br>(9 ± 3)
IL6<br>IL-1β<br>TNF-α<br>TIMP-1	**CB**	103.4 ± 19.37<br>(71.67 ± 20.88)	62.4 ± 8.195<br>(56.17 ± 18.16)	41 ± 11.34<br>(21.4 ± 3.53)
	**mPB**	52.25 ± 11.69<br>(22 ± 8.07)	26.25 ± 6.156<br>(6.33 ± 2.33)	26 ± 8.175<br>(9 ± 3)
